# Molecular Dynamics Insights into Bio-Oil-Enhanced Self-Healing of Aged Asphalt

**DOI:** 10.3390/ma18153472

**Published:** 2025-07-24

**Authors:** Liuxiao Chen, Silu Tan, Mingyang Deng, Hao Xiang, Jiaxing Huang, Zhaoyi He, Lin Kong

**Affiliations:** 1 Shock and Vibration of Engineering Materials and Structures Key Laboratory of Sichuan Province, Southwest University of Science and Technology, Mianyang 621010, China; 2 College of Civil Engineering and Architecture, Southwest University of Science and Technology, Mianyang 621010, China; 3 National & Local Joint Engineering Research Center of Transportation and Civil Engineering Materials, Chongqing Jiaotong University, Chongqing 400074, China; 4 Survey and Design Company of Sichuan Road & Bridge (Group) Co., Ltd., Chengdu 610041, China; 5 College of Civil Engineering, Southwest Jiaotong University, Chengdu 610031, China

**Keywords:** bio-oil-recycled asphalt, molecular structure, self-healing, mean square displacement, molecular diffusion

## Abstract

Long-term aging deteriorates asphalt’s self-healing capacity, yet the molecular mechanisms of bio-oil rejuvenation remain unclear. The fluidity and healing index of an asphalt binder were tested using a dynamic shear rheometer, and a healing model was established using molecular dynamics software to analyze the movement state. The results show that after adding the bio-oil, the healing index of aged asphalt increases significantly, lowering the optimal healing temperature by 10.1 °C. MD simulations demonstrate that bio-oil weakens van der Waals forces (with a 15.3% reduction in non-bonded energy) to enhance molecular diffusion, with a critical healing distance of 0.87 Å and aggregation at 1.11 Å. The bio-oil reduces the activation energy for healing from 4.97 kJ/mol (aged asphalt) to 3.75 kJ/mol. Molecular dynamics simulations can effectively aid scholars in understanding the asphalt healing process and movement patterns.

## 1. Introduction

Under the long-term effects of traffic loads and the external environment, asphalt pavements are susceptible to microcracks that develop into macrocracks [1]. According to Bazin [2], the self-healing properties of asphalt materials allow the repair of cracks. Therefore, relevant studies have received considerable attention. However, during the service life of asphalt, aging is inevitable, leading to a gradual deterioration of its self-healing performance [3]. This decline is attributed to changes in the content of various components within the aged asphalt [4]. In recent years, bio-oil, an effective and environmentally friendly rejuvenator, has been introduced into aged asphalt to compensate for the oil loss caused by aging, thereby improving its road performance [5], particularly its self-healing capability [6].

Scholars commonly use dynamic shear rheometer (DSR) tests to evaluate the rheological properties of bio-oil-rejuvenated asphalt, assessing its self-healing ability based on the recovery of complex moduli and related indices. The self-healing capacity of aged asphalt is enhanced after bio-oil treatment, yet the efficacy varies significantly among different bio-oil types [7,8].

Bio-oil rejuvenation of aged asphalt is a physical process [9]. Although bio-oil-rejuvenated asphalt exhibits a notable capacity to reverse microdamage, this behavior occurs at the nano-scale. The information obtained from macroscopic tests is insufficient to reveal the self-healing mechanism of asphalt. Therefore, understanding the self-healing behavior of asphalt materials at the nano-scale is of significant importance [10].

Molecular dynamics (MD) simulations can be employed to model the impact of bio-oil rejuvenators on the properties of aged asphalt, revealing the diffusion [11] and aggregation [12] behaviors of the rejuvenated asphalt components. MD simulations are suitable for predicting asphalt self-healing, with accurate molecular structures forming the basis for these simulations [13]. Parameters such as molecular concentration, energy changes, and diffusion characteristics during the self-healing process of the asphalt model can provide additional insights into the self-healing mechanism of cracks [14]. Components with lower molecular weights and elongated molecular structures are conducive to asphalt self-healing [15]. Zhang [16], Xu [17], and Zheng [18] used residual soybean oil and vegetable oils to rejuvenate aged asphalt binders, finding that light oils reduced the energy barrier for asphalt reactions and enhanced the self-healing potential. Bio-oil rejuvenators outperform petroleum-based rejuvenators in restoring the self-healing ability of aged asphalt [19]. The liquefaction residue of straw is beneficial for the self-healing of regenerated asphalt, and the higher the content, the greater the diffusion coefficient [20]. Ruan [12] used an MD method to evaluate the effect of linoleic acid and oleic acid molecules on aging asphalt binder molecules.

Scholars worldwide have successively conducted research on the self-healing of bio-oil-rejuvenated asphalt, primarily focusing on characterization methods and the factors influencing self-healing performance [21,22]. However, studies on the self-healing mechanisms of bio-oil-rejuvenated asphalt are relatively scarce, and there is also a lack of comparative analyses between laboratory tests and numerical simulation results. Consequently, this work uses linseed oil as a representative bio-oil and 70# asphalt to perform DSR experiments coupled with MD simulations. A quantitative model linking the MD-derived diffusion coefficients to experimental healing indices is established, while the activation energy calculations elucidate how bio-oil molecular structures lower the energy barrier for the self-healing of aged asphalt. The findings provide a theoretical basis for screening diverse bio-oil rejuvenators and designing low-temperature self-healing asphalt pavements.

## 2. Materials and Methods

### 2.1. Asphalt and Regeneration Agent

In the experiment, 70# asphalt binder, with a needle penetration of 69 dmm at 25 °C, a softening point of 53 °C, and an elongation of more than 150 mm at 15 °C, was used. Rotating film oven and pressure-aging experiments were conducted to simulate asphalt aging. The needle penetration of the aged asphalt was 24 dmm, the softening point was 67 °C, and the elongation was 5 cm at 15 °C. The testing methods referred to the current Chinese specification “Standard Test Methods of Bitumen and Bituminous Mixtures for Highway Engineering” (JTG E20—2011) [23].

A bio-oil regeneration agent was prepared using linseed oil as the base. The main components were unsaturated fatty acids. The viscosity was 0.18 Pa·s at 60 °C, the flash point was 270 °C, the mass loss before and after aging in the rotating film oven was 2.3%, the viscosity ratio was 0.94, and the relative density was 0.93. Furthermore, the aged asphalt was pre-heated to 150 °C, the bio-oil was slowly poured under 800 rpm shear for 15 min, and the blend was kept at 150 °C for another 30 min to ensure homogeneity. A 7% bio-oil regeneration agent (mass fraction) was added to the aged asphalt to prepare the bio-oil-recycled (BR) asphalt, with a needle penetration of 58 dmm, a softening point of 55 °C, and an elongation of 52 cm.

### 2.2. Dynamic Shear Rheometer Test

The dynamic shear rheometer experiment used the Discovery series DHR-2 dynamic shear rheometer from the TA Company in New Castle, DE, USA. The asphalt flow behavior was analyzed using the frequency scanning mode. The frequency scanning range was 0.1–10 Hz, the control strain was 0.1%, the parallel plate size was 25 mm, the parallel plate spacing was 2 mm, the scanning temperature was 30–70 °C, and the interval temperature was 10 °C. The composite viscosity of asphalt and shear frequency can be fitted using Equation (1) [24], and the relationship between the flow performance of asphalt and temperature can be obtained as follows:

(1) $$\eta^{*}=m{\left|\omega \right|}^{n-1}$$

,

where ω denotes the shear frequency; $\eta^{*}$ denotes the complex viscosity; m and n denote the fitting parameters, and n is termed as the flow behavior index.

### 2.3. Molecular Model of Asphalt Components

**Figure 1:**
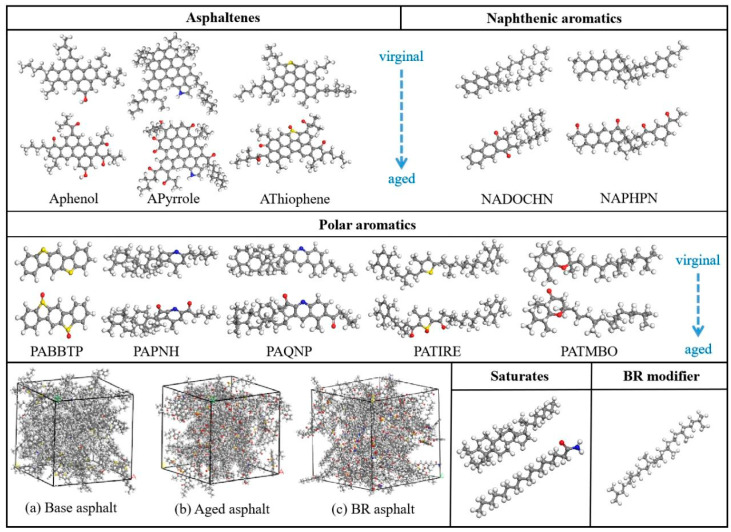
Molecular structures of asphalt components.

In this study, the aged asphalt model was characterized by the addition of sulfoxide and carbonyl groups. Bio-oils contain many amides and nitrogen-containing functional groups, and a typical straight-chain molecular structure was selected for the characterization [25,26]. Figure 1 shows the molecular structures of the asphalt components and the bio-oil regeneration agents.

Based on this model, an asphalt molecular model was established. Table 1 lists the contents of each model component. Asphaltenes, polar aromatics, saturates, and naphthenic aromatics in the base asphalt comprised 17.53%, 38.75%, 11.29%, and 32.43% of the total content, respectively. In the aged asphalt model, the content above constituted 24.83%, 43.42%, 11.32%, and 20.43% of the total, respectively. In the BR asphalt model, the content above constituted 23.12%, 40.40%, 10.49%, and 19.01% of the total, respectively. Additionally, the mass fraction of the bio-oil regenerant was 6.98%. It was annealed 20 times via the NVT system synthesis in the Forcite module. The model with the lowest energy and the most stable structure was selected after 20 annealing cycles.

### 2.4. Molecular Model of Healing

**Figure 2:**
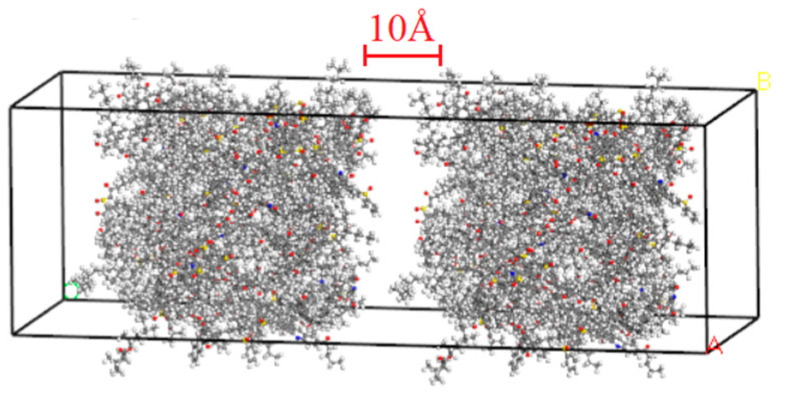
Asphalt healing model with initial crack.

Optimized and annealed asphalt models were used as the bases for this study. A layer vacuum was established using a built-in layer module to develop an asphalt healing model with microcracks. Figure 2 shows an asphalt healing model with a crack size of 10.0 Å. We used dynamics in the Forcite Calculation module for the calculation, in which the task module selects the Condensed-phase Optimized Molecular Potentials for Atomistic Simulation Studies (COMPSS) and the ensemble module selects the canonical ensemble mode with a time step of 1 fs, a total simulation time of 100 ps, NPT mode, and 0.0001 GPa. To simulate asphalt healing at different ambient temperatures, the healing temperatures were set as 273, 298, 323, 348, and 373 K.

## 3. Analysis of Experimental Results

### 3.1. Dynamic Shear Rheometer Scan Results

**Figure 3:**
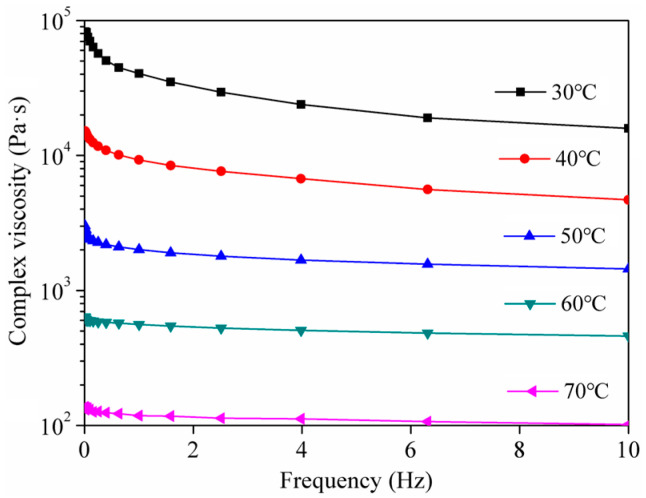
Composite viscosity of asphalt at different temperatures.

The relationship between the complex viscosity and the frequency of 70# asphalt is shown in Figure 3. At the same frequency, as the temperature increased, the complex viscosity of the asphalt decreased significantly. At the same temperature, as the frequency increased, the complex viscosity decreased. When the temperature exceeded 50 °C, the difference in the viscosity change decreased, and the asphalt complex viscosity–shear frequency curve tended to be stable.

**Figure 4:**
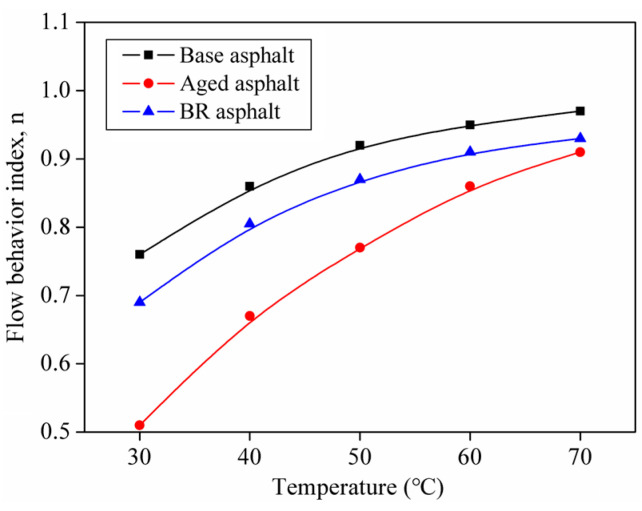
Flow behavior index of different asphalts.

The relationship between the temperature and the asphalt flow behavior index can be obtained by fitting the asphalt composite viscosity and shear frequency according to Equation (1) (Figure 4).

Asphalt aging led to a significant decrease in the flow behavior index; however, this decrease was attenuated at high temperatures. After adding the bio-oil regenerant, the flow behavior index of the aged asphalt was restored and the flow performance of the aged asphalt was improved.

When the flow behavior index is 1, the material behaves as a Newtonian fluid. When the flow behavior index is n ≥ 0.9, the material approaches a near-Newtonian fluid state, demonstrating good flow properties [27]. Thus, the temperature at which n = 0.9 is identified as the potential optimal healing temperature for asphalt. At this temperature, the asphalt flows spontaneously and exhibits favorable self-healing characteristics. For the base asphalt, BR asphalt, and aged asphalt, the temperatures corresponding to n = 0.9 were 46.9 °C, 58.2 °C, and 68.3 °C, respectively. This indicates that the addition of a bio-oil rejuvenating agent can lower the temperature required for aged asphalt to achieve a near-Newtonian fluid state. At higher temperatures, asphalt displays a near-Newtonian fluid behavior that allows it to quickly wet and close cracks, facilitating the diffusion of interface molecules to build strength and accelerate the self-healing of asphalt [3].

**Figure 5:**
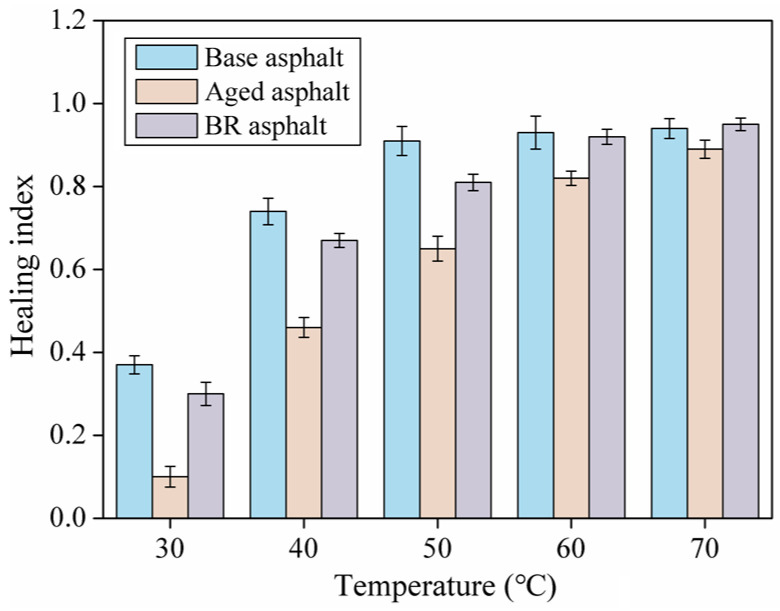
Healing index of asphalts at different temperatures.

As the healing temperature increased, the healing indices of the different asphalts increased continuously, as shown in Figure 5.

After adding the regeneration agent, the healing index of the aged asphalt increased significantly, and the relationship curve between the healing index of the aged asphalt and the healing temperature approached that of the matrix asphalt, indicating that the regeneration agent can restore the healing ability of the aged asphalt. This is because the regeneration agent can supplement the light components missing in the aged asphalt [28], dilute and disperse the strong polar substances in the aged asphalt, restore the colloidal structure of the aged asphalt, improve the fluidity, and improve the self-healing performance.

### 3.2. Molecular Dynamics Simulation Results

#### 3.2.1. Validation of Asphalt Molecular Model

**Figure 6:**
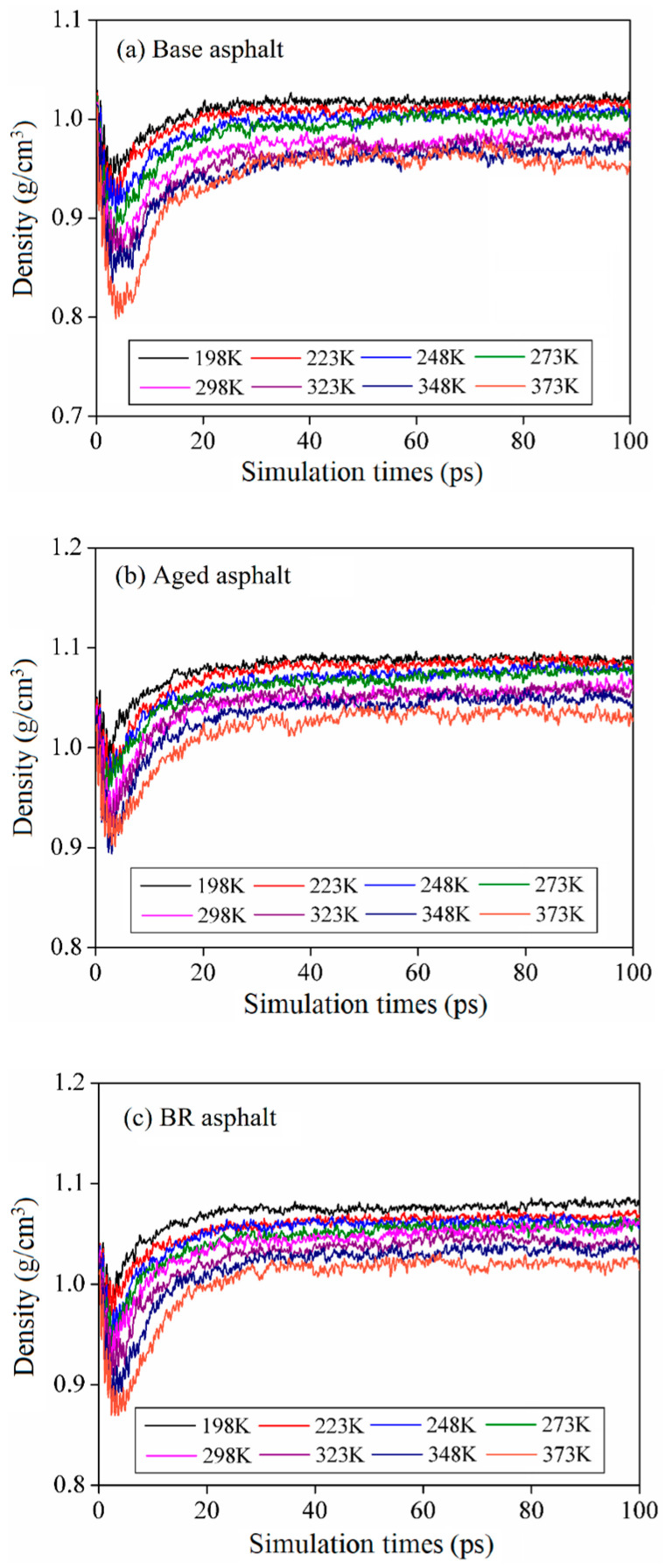
Density of asphalt molecular models at different temperatures.

Density calculations were performed in the dynamic mode of the Forcite module. A COMPASS force field of 0.0001 GPa was applied [29]. The asphalt molecular model densities were calculated at 198, 223, 248, 273, 298, 323, 348, and 373 K. The calculation time was set to 100 ps. Figure 6 shows the calculated density of the asphalt molecular model after 20 ps.

The density indicated by the models fluctuated slightly with time but decreased significantly as the temperature increased. The average density value in the stable-stage range was selected to characterize the model density. The density results are listed in Table 2. Under the same conditions, the density of the aged asphalt exceeded that of the base asphalt. Given the bio-oil regenerant, the density of the BR asphalt was lower than that of the aged asphalt. The model density is consistent with the actual situation [3].

**Figure 7:**
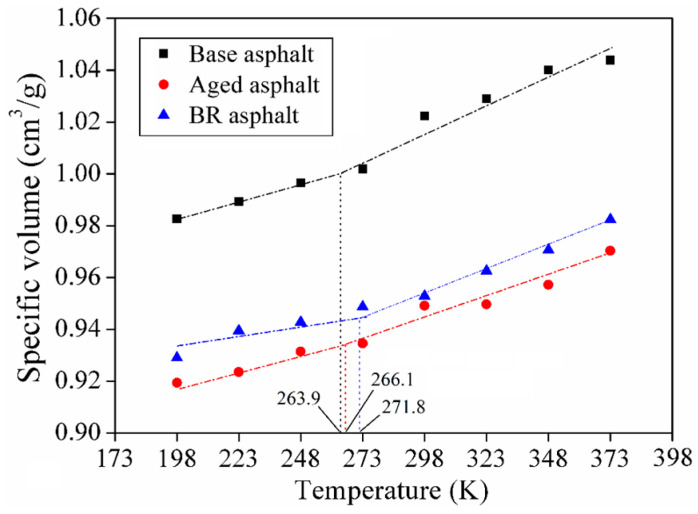
Specific volume of asphalt molecular models.

Based on free volume theory, the polymer volume was segmented into the actual molecular volume and the molecular interstitial volume, which are known as the occupied volume and free volume, respectively. When the ambient temperature was below the glass transition temperature, the free volume remained nearly constant. When the ambient temperature was above the glass transition temperature, motion was triggered in the polymer chain segment, and the free volume expansion resulted in a change in the polymer volume expansion rate. Figure 7 shows the glass transition temperature obtained from the abrupt change in specific volume. The glass transition temperatures of the base, aged, and BR asphalts were −9.1 °C, −1.2 °C, and −6.9 °C, respectively, which were consistent with the measured values [30]. The asphalt molecular model was confirmed to be reliable based on the density and glass transition temperature.

#### 3.2.2. Radial Distribution Function

**Figure 8:**
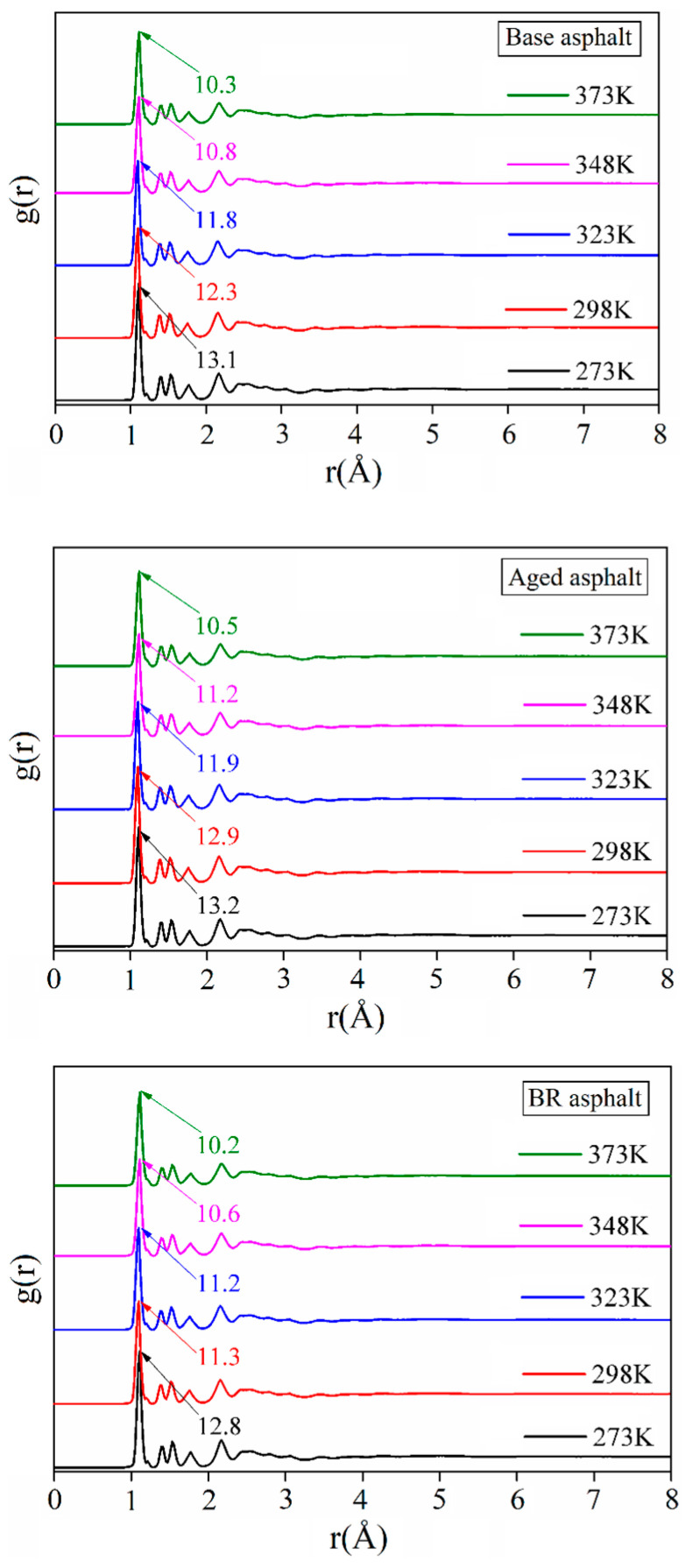
Radial distribution function of asphalt healing models.

The radial distribution function (RDF) reflects the distribution and aggregation of molecules within a material. Figure 8 shows the radial distribution function of the asphalt healing model at different temperatures. Multiple sharp oscillation peaks appeared between 0.87 Å and 3 Å, which indicates that the minimum distance for asphalt model healing is more than 0.87 Å. The maximum peak was achieved at 1.11 Å, which indicates the occurrence of aggregation or stacking.

Aging enhances molecular aggregation in asphalt, with an average peak increase of 2.5%. Bio-oil regenerant molecules can effectively reduce the aggregation of aged asphalt molecules, with an average peak reduction of 5.8%. The amplitude of the oscillation decreased between 3 Å and 5 Å. It gradually smoothed and stabilized after 5 Å. This indicates that the asphalt molecules are randomly distributed at this distance, and that the intermolecular interactions are dominated by van der Waals forces [31].

#### 3.2.3. Size and Density

**Figure 9:**
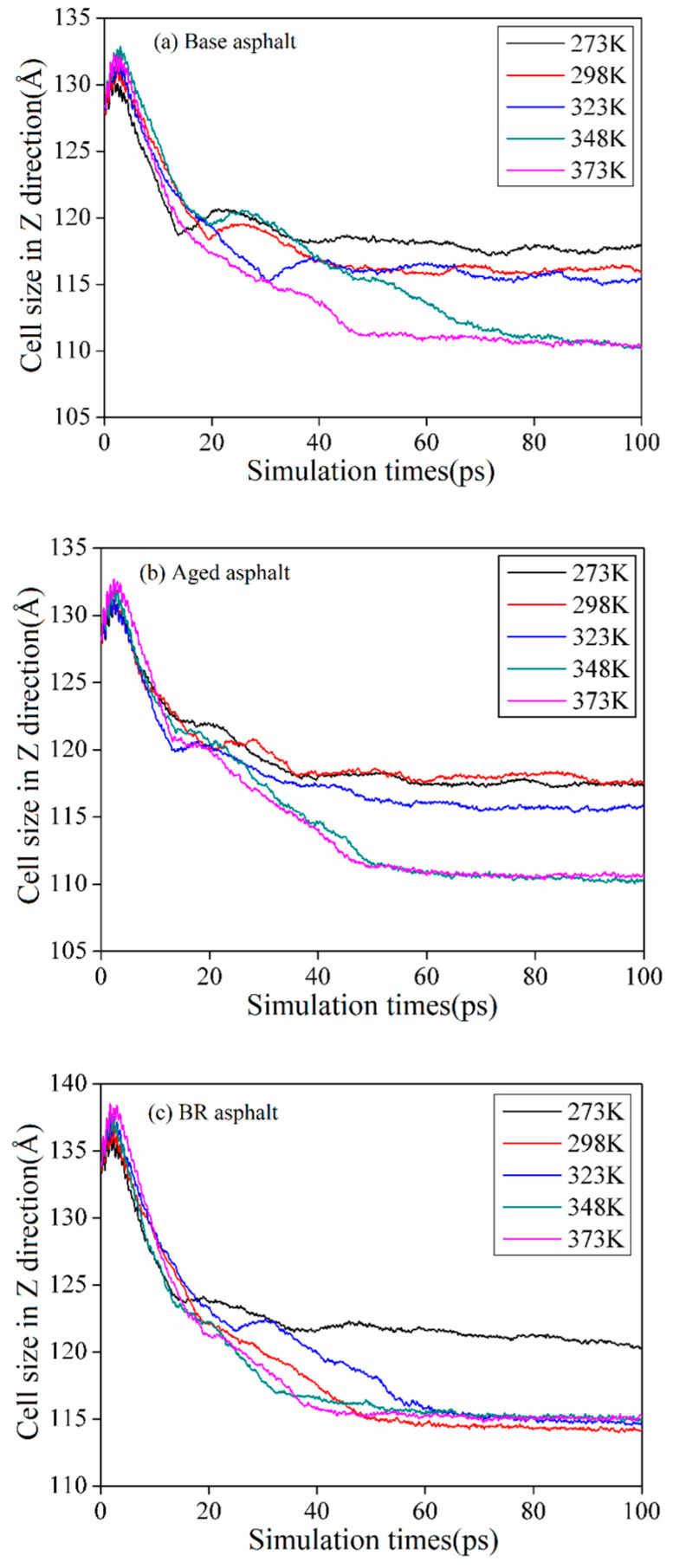
Cell size of asphalt healing models.

The cell size gradually changed with the molecular movement. Figure 9 shows the size of the cells along the *z*-axis. In general, the cell size of the asphalt healing model decreased gradually in the early stages of healing and then stabilized after 30 ps. This is consistent with the results of previous studies [32]. This implies that the asphalt molecules continue to approach each other to occupy a predetermined vacuum crack position and facilitate the disappearance of the crack, which describes the microscopic process of internal crack healing.

**Figure 10:**
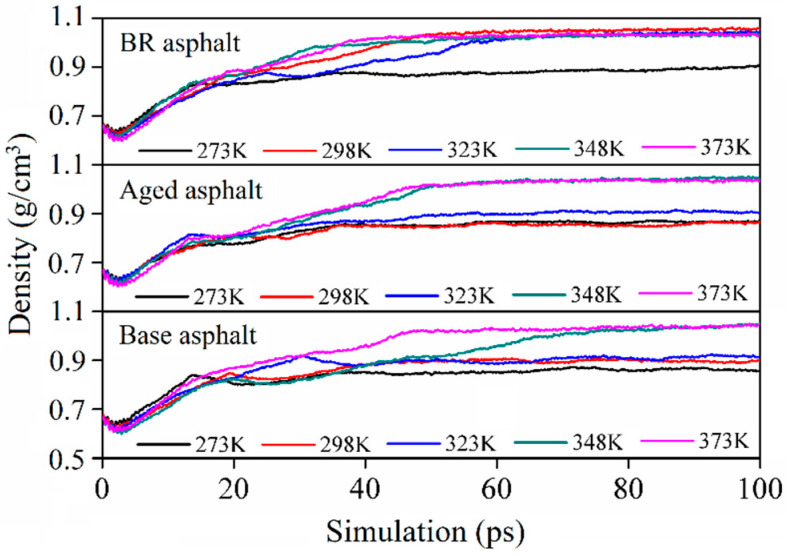
Density of asphalt healing model.

Figure 10 shows that, at temperatures in the range of 273–323 K, the density of the healing model of the recycled asphalt was significantly higher than that of the aged asphalt. When the temperature exceeded 323 K, the density difference between the three asphalt healing models decreased.

#### 3.2.4. Energy Distribution

During the healing process, asphalt molecules are in constant motion. If thermodynamic statistics are performed on the microscopic motion processes and states of all molecules, physical quantities that reflect the macroscopic characteristics of the system can be obtained, which mainly include potential energy, kinetic energy, non-bonded energy, and total energy [33].

**Figure 11:**
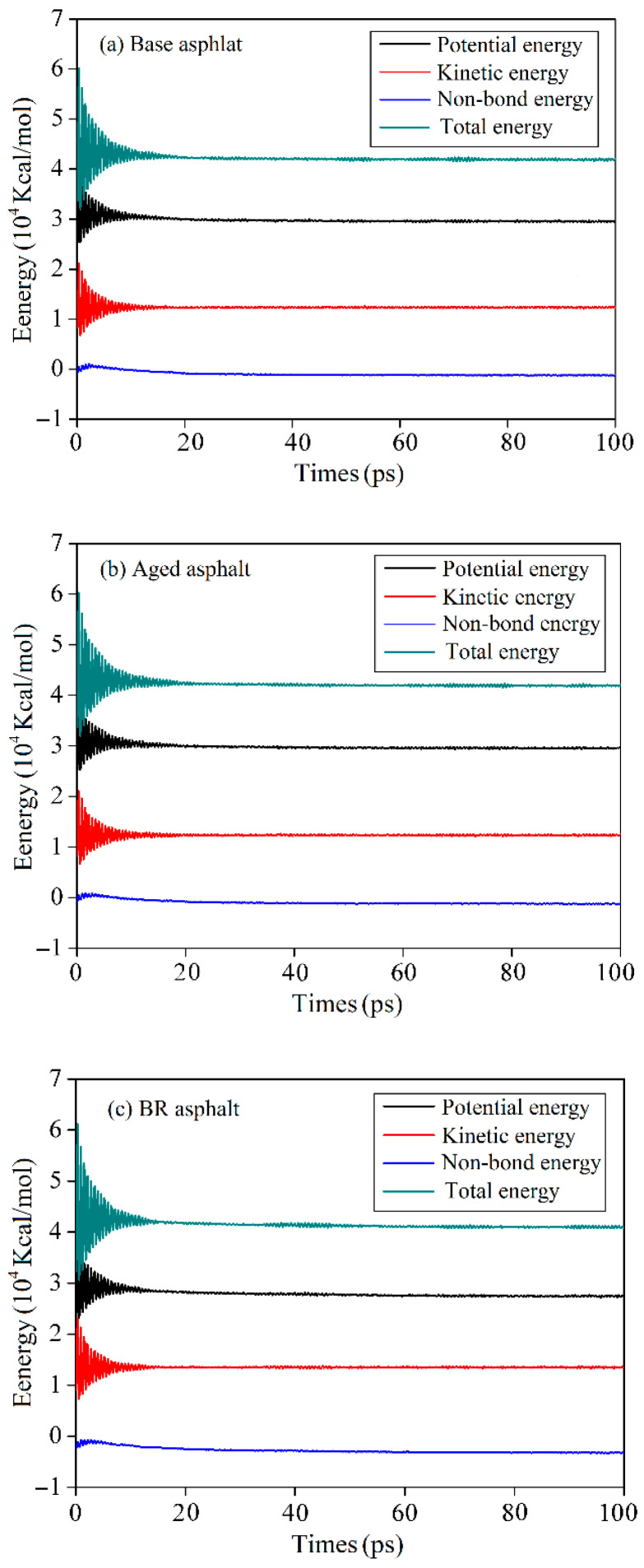
Energy distribution of asphalt healing models.

Figure 11 shows the variation in energy with healing time for asphalt at 298 K. The results showed that energy continued to decrease, accompanied by significant fluctuations at the beginning of the healing process. The fluctuation–reduction phase was completed within 20 ps, followed by a continuous stabilization phase. The decrease in the non-bonding energy during the self-healing process suggests that the self-healing process is dominated by intermolecular forces [32]. As the electrical energy of the intermolecular forces remains unchanged, the van der Waals forces decrease significantly. These results indicate that the van der Waals forces of the asphalt self-healing process significantly affect healing.

The energy distribution of the healing model varies with respect to healing temperatures. Table 3 lists the average values of the energy parameters of the asphalt healing model after the stabilization phase. The overall energy change trend remained unchanged as the temperature increased. At higher temperatures, the potential, kinetic, and total energies were higher, whereas the non-bonding energies were lower. This indicates that high temperatures enhance molecular motion and promote the asphalt healing process.

#### 3.2.5. Mean Square Displacement

The molecules move continuously during the asphalt healing process. The difference between the motion distance of a particle at the initial and calculated moments is defined as the mean square displacement (MSD), which effectively reflects the motion of a molecule [34]. Equation (2) expresses the formula for calculating the mean square displacement.

(2) $$MSD(t)={\langle r_{i}(t)-r_{i}(0)\rangle }^{2}$$

,

where MSD(t), $r_{i}(0)$, and $r_{i}(t)$ are the mean square displacements of the particle and motion vector positions at the initial and calculation moments, respectively.

**Figure 12:**
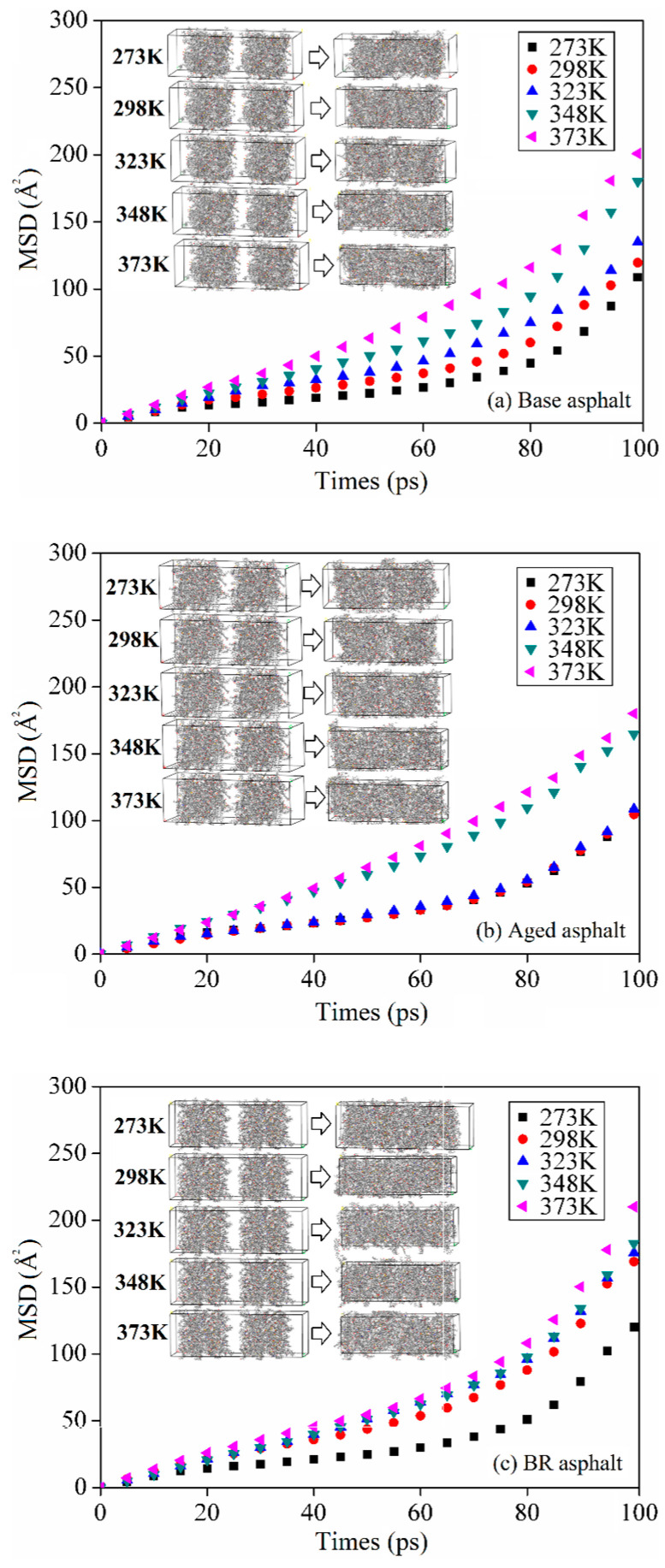
Mean square displacement during asphalt model healing.

The mean square displacement increased gradually with the healing time, as shown in Figure 12. The curve can be categorized into two phases based on the growth rate. The first was the slow-growth phase, which occurred 40 ps prior to the onset of healing. The small slope increased and the mean square displacement increased smoothly in this stage. This implies that the entire healing system was in the adaptation and relaxation phases, whereas the atoms were limited by displacement. The rapid-growth phase occurred after 40 ps of healing, which implied that the healing system had transitioned to optimal atomic motion [35].

Compared with the mean square displacement at 273 K, the mean square displacement of the base, aged, and BR asphalts increased by 92.2, 74.1, and 90.2 Å2 within 100 ps at 373 K, respectively, which corresponded to an increase of 84.8%, 69.9%, and 75.2%, respectively. This indicates that high temperatures promoted asphalt healing. Under the same conditions, the mean square displacement of the aged asphalt was significantly smaller than that of the base asphalt. After the addition of the bio-oil regenerant, it improved to a level similar to that of the base asphalt. This indicates that the bio-oil regenerants can promote the movement of asphalt molecules and repair cracks. These conclusions are consistent with existing studies [14,20].

#### 3.2.6. Diffusion Coefficient

The repair behavior of cracks can also be regarded as a result of molecular diffusion and filling [36]. The diffusion coefficient, which is a particle diffusion rate parameter, can be used to quantitatively characterize the self-healing ability of asphalt. According to Fick’s law (Equation (3)), the diffusion coefficient is determined by the material type and the external environment. Substituting Equation (2) into Equation (3) results in an expression for the diffusion coefficient, as shown in Equation (4).

(3) $$D=\frac{1}{6}\underset{t\to \infty }{\lim}\frac{d}{dt}\sum_{i=1}^{N}\left\{{\left[r_{i}\left(t\right)-r_{i}\left(0\right)\right]}^{2}\right\}$$

(4) $$D=\frac{m}{6},$$

where D, $r_{i}\left(0\right)$, and $r_{i}\left(t\right)$ denote the diffusion coefficient and motion vector positions at the initial and calculation moments, respectively.

**Figure 13:**
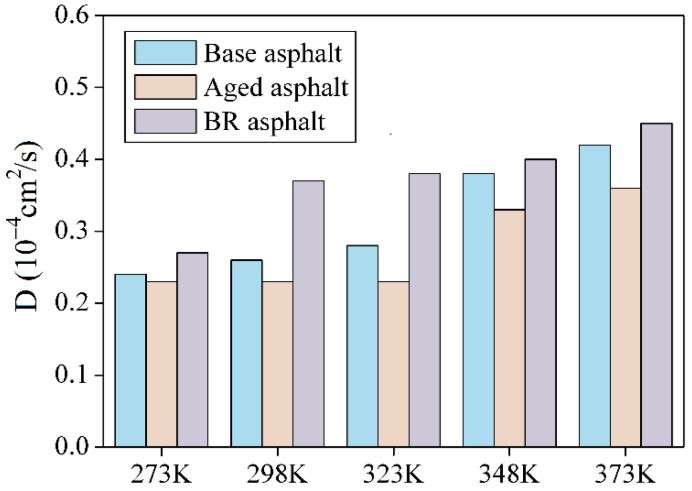
Diffusion coefficient of asphalt molecules at different temperatures.

Figure 13 shows that, at high temperatures, the diffusion rate of the asphalt molecules increased. Compared with the diffusion coefficients at 273 K, the diffusion coefficients of the base, aged, and BR asphalts at 373 K increased by 18 × 10^−6^ cm^2^/s, 14 × 10^−6^ cm^2^/s, and 18 × 10^−6^ cm^2^/s, respectively, which corresponded to an increase of 74.8%, 60.2%, and 64.6%, respectively.

Laidler [37] similarly concluded that the diffusion coefficient is closely associated with the ambient temperature. Subsequently, Sun [38] applied the Arrhenius law to express the diffusion coefficient as a function of the temperature in an asphalt model, as shown in Equations (5) and (6), respectively.

(5) $$D=A\exp(-\frac{E}{RT})$$

(6) $$\ln(D)=\ln(A)-\frac{E}{R}\cdot \frac{1}{T},$$

where A denotes the exponential factor, E denotes the activation energy, R denotes the gas constant (with a value of 8.314 J/mol/K), and T denotes the ambient temperature.

**Figure 14:**
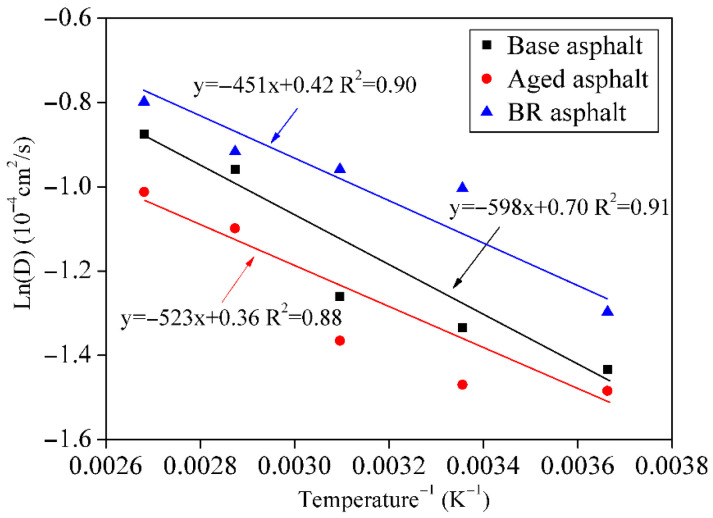
Relationship between ln (D) and 1/T.

A graph of ln(D) versus 1/T is shown in Figure 14. The exponential factor and activation energy were obtained from the intercept and slope of the linear fitting equation, respectively. Activity energy can be regarded as the energy required by the asphalt molecules to initiate self-healing. The exponential factor characterizes the instantaneous stress obtained during the instantaneous healing of a cracked interface [36].

The activation energy required for the BR asphalt was 3.75 kJ/mol, which was less than the activation energy required for the aged asphalt (4.97 kJ/mol). However, the exponential factor for the former was 1.52 cm^2^/s, and the exponential factor for the latter was 1.43 cm^2^/s. This indicates that the intermolecular stress is greater during the instantaneous healing of the BR asphalt and that the aged asphalt requires more energy to achieve healing.

## 4. Conclusions

This study investigated the self-healing capability of bio-oil-rejuvenated asphalt through dynamic shear rheological testing and molecular dynamics simulations. It was concluded that the asphalt flow behavior index first increased rapidly and then slowly with an increase in temperature. The healing index decreased after asphalt aging, and the healing index improved after bio-oil regeneration. The radial distribution function curves of the asphalt healing model were similar under all conditions. In particular, g (r) was 0 within 0.87 Å, and mutual attraction between molecules began after 0.87 Å. Peak g (r) reached a maximum at 1.11 Å, where molecular aggregation or stacking occurred.

As the healing temperature increased, the potential, kinetic, and total energy of the healing model increased, whereas the non-bonding energy decreased. The energy fluctuations stabilized after 20 ps. The mean square displacement and diffusion coefficient increased with the healing temperature. The BR asphalt had greater transient stress during healing, whereas the aged asphalt required more energy to achieve healing.

Overall, these findings offer new perspectives and methodologies for analyzing the self-healing capabilities of bio-oil-rejuvenated asphalt. However, considering the diversity of bio-oils and the potential impacts of real-world factors (moisture, traffic cycles, long-term aging) on self-healing, we recommend future work using accelerated aging tests and multiphysics simulations. Additional experimental data will further enrich the research in this field.

## Figures and Tables

**Table 1:** Types of asphalt and component content.

Components	Molecular Formula	Number of Atoms	Mass Percent (%)		
			Base Asphalt	Aged Asphalt	BR Asphalt
Squalane	C_30_H_62_	92	5.27	4.78	4.4
SHopane	C_35_H_62_	97	6.02	6.54	6.09
NAPHPN	C_35_H_44_	79	15.94	10.59	9.85
NADOCHN	C_30_H_46_	76	16.49	9.84	9.16
PAQNP	C_40_H_59_N	100	5.18	7.89	7.34
PATIRE	C_40_H_60_S	101	7.15	8.36	7.78
PATMBO	C_29_H_56_O	86	6.56	6.87	6.39
PAPNH	C_36_H_57_N	94	6.28	7.2	6.70
PABBTP	C_18_H_10_S_2_	30	13.58	13.1	12.19
APhenol	C_42_H_54_O	97	5.38	7.12	6.63
APyrrole	C_66_H_81_N	148	5.54	8.91	8.30
AThiophene	C_51_H_62_S	114	6.61	8.8	8.19
BR	C_16_H_33_NO	51	/	/	6.98

**Table 2:** Average density of asphalt molecular models during the stable phase.

Type	198 K	223 K	248 K	273 K	298 K	323 K	348 K	373 K
Base asphalt	1.018	1.011	1.004	0.998	0.978	0.972	0.961	0.958
Aged asphalt	1.088	1.083	1.074	1.070	1.054	1.053	1.045	1.031
BR asphalt	1.076	1.064	1.061	1.054	1.050	1.039	1.030	1.018

**Table 3:** Energy parameters of asphalt healing models.

ID	Energy	273 K	298 K	323 K	348 K	373 K
Base asphalt	Potential (Kcal/mol)	28,556	29,632	30,810	31,867	32,874
	Kinetic (Kcal/mol)	11,313	12,348	13,389	14,423	15,458
	Non-bond (Kcal/mol)	−1233	−1139	−937	−855	−826
	Total (Kcal/mol)	39,869	41,981	44,200	46,290	48,333
Aged asphalt	Potential (Kcal/mol)	28,530	29,650	30,780	31,770	32,890
	Kinetic (Kcal/mol)	11,316	12,354	13,389	14,425	15,457
	Non-bond (Kcal/mol)	−1252	−1118	−1007	−941	−820
	Total (Kcal/mol)	39,846	42,004	44,170	46,196	48,347
BR asphalt	Potential (Kcal/mol)	26,520	27,678	28,969	30,115	31,303
	Kinetic (Kcal/mol)	12,393	13,527	14,657	15,794	16,932
	Non-bond (Kcal/mol)	−3067	−2998	−2802	−2709	−2616
	Total (Kcal/mol)	38,914	41,206	43,626	45,910	48,236

## Data Availability

The original contributions presented in this study are included in the article. Further inquiries can be directed to the corresponding author.
